# Outcomes of operative and non-operative management of acromial and scapular spine fractures after reverse shoulder arthroplasty: a systematic review

**DOI:** 10.1016/j.jsea.2026.100081

**Published:** 2026-08-12

**Authors:** Amin Abukar, Cassandra Case, Ujash Sheth, Patrick Henry, Diane Nam

**Affiliations:** Department of Orthopedic Surgery, University of Toronto, Sunnybrook Orthopaedic Upper Limb (SOUL), Sunnybrook Health Sciences, Toronto, ON, Canada

**Keywords:** Acromial stress fracture, Acromial fracture, Scapular spine fracture, Reverse total shoulder arthroplasty, Reverse total shoulder replacement, ASF, rTSA

## Abstract

**Background:**

Reverse total shoulder arthroplasty (rTSA) use is rising. Acromial stress fractures (ASFs) are infrequent but significant complication associated with pain and functional limitation. Optimal management remains uncertain. This review compared union, functional outcomes, and complication between non-operative and operative management of ASF post-rTSA.

**Methods:**

Systematic review was conducted in accordance with Preferred Reporting Items for Systematic Reviews and Meta-Analyses guidelines. MEDLINE, Embase, Scopus, Web of Science, and the Cochrane Library were searched. Randomized controlled trials, observational studies, and case series including ≥3 adult patients with post-rTSA ASFs reporting management and outcomes were eligible. A descriptive quantitative synthesis was undertaken.

**Results:**

Fourteen studies (376 fractures in 374 patients; mean age 72.9 years; 78% female) were included. ASF incidence (from 4 studies) ranged from 3.1 to 7.2%. Seventy percent of fractures occurred atraumatically; mean time to diagnosis was 11.3 months; mean follow-up was 41.1 months. Levy type II predominated. Management was non-operative (73.9%) or operative (26.1%). Overall union was 68%, higher after fixation (78% vs. 59.8%; *P* = .004). Patient-reported outcomes differed modestly: visual analog scale 1.7 versus 2.4, American Shoulder and Elbow Surgeons 66.9 versus 60.2, Constant score 37.9 versus 46.0, and Subjective Shoulder Value 63.5 versus 60.7 (operative vs. non-operative). External rotation trended higher after surgery. Operative complications occurred in 28.2%, with 91% requiring reoperation, most hardware irritation or fixation failure.

**Conclusion:**

ASFs post-rTSA remains challenging. Surgical fixation demonstrates higher union, but functional outcomes are comparable to non-operative management and surgery carries notable complication burden. Given the descriptive, Level IV evidence, findings are hypothesis-generating; treatment should be individualized, and prospective comparative studies using standardized classifications/outcomes are required.

Globally, reverse total shoulder arthroplasty (rTSA) has continued to gain popularity, with expanding indications now accounting for 70% of all primary shoulder arthroplasty in certain regions.^20^ However, post-rTSA changes in shoulder biomechanics can result in long-term complications such as acromial fractures or fractures of the scapula spine collectively referred to as acromial stress fractures (ASFs). The hypothesized mechanism is stress concentration at the deltoid origin secondary to changes in lever arm of the deltoid, baseplate screws acting as stress-risers and repeated abutment on the acromion.^7^ The reported incidence of ASFs ranges from 0.8% to 11%, and these fractures remain challenging to manage.^1^

Several risk factors have been identified that may predispose to ASF. These include older patients with lower BMI, osteoporosis, inflammatory arthritis, female sex, previous rotator cuff repair, and technical factors which include increased distalization and lateralization.^7^^,^^14^ Two main classification systems are used for ASF, namely the Crosby and Levy (see Table I). The Crosby classification system centers on relationship of fracture line to the AC joint, with Crosby type 1 fractures being avulsion from anterior acromion, type 2 are anterior acromion fractures that start just posterior to AC joint and type 3 are posterior acromion or scapular spine fractures. The Levy classification centers on the anatomic location of deltoid origin, with Levy type 1 fractures involving a portion of portion of anterior and middle deltoid origin, type 2 fractures involving the entire middle deltoid and portion of posterior deltoid origin, while type 3 fractures involve the entire middle deltoid and posterior deltoid origin.^4^^,^^17^

**Table I:** Summary of ASF classification.

Classification system	Basis of classification	Type	Anatomic description
Crosby^4^	Location of the fracture line relative to the acromioclavicular (AC) joint	I	Avulsion fracture of the anterior acromion
		II	Acromial fracture beginning just posterior to the AC joint
		III	Fracture of the posterior acromion or scapular spine
Levy^17^	Extent of deltoid-origin involvement (anatomic location)	I	Involves a portion of the anterior and middle deltoid origin
		II	Involves the entire middle deltoid origin and a portion of the posterior deltoid origin
		III	Involves the entire middle and the entire posterior deltoid origin
Seebauer^9^	Anatomic location of the fracture	I–III	Modified Levy classification to distinguish between anterolateral and posterolateral acromial fractures (type IIA and IIB) and introduced trans-sagittal scapular spine fracture (type IIIB).

*ASF*, acromial stress fracture; *AC*, acromioclavicular.

Patients who develop ASF post-rTSA have been shown to experience inferior functional outcomes than those without fractures. However, it remains unclear which patients benefit more from conservative compared with operative management.^8^ Conservative management usually involves shoulder immobilization followed by progressive range of motion. Operative management involves open reduction and internal fixation, and a variety of options have been reported in the literature, such as tension band construct, dual plating, and mesh plating.^10^^,^^13^ Two recent systematic reviews have specifically examined operative treatment and fixation constructs for these injuries^3^^,^^12^; however, a synthesis that directly contrasts operative and non-operative pathways across union, functional, and complication domains remain limited.

The current literature presents conflicting evidence regarding whether conservative treatment is non-inferior to surgical fixation. A survey in 2011 of the American Shoulder and Elbow Surgeons (ASES) members revealed the majority favored conservative treatment for ASFs. Conversely, some reports suggest that surgically managed patients may demonstrate improvement relative to their post-fracture baseline.^5^^,^^21^

Despite increasing interest in recent years, there remains no clear consensus regarding the optimal management of ASFs following rTSA. The primary aim of this systematic review was to evaluate the available evidence and compare reported union rates, functional outcomes, and complication profiles between conservative and operative management strategies for post-rTSA ASFs.

## Materials and methods

### Search strategy

This systematic review was conducted in accordance with the Preferred Reporting Items for Systematic Reviews and Meta-Analyses guidelines. A comprehensive search was performed using Embase, MEDLINE, Scopus, Web of Science, and Cochrane Library. Preliminary search terms related to fracture, scapula, acromion, and rTSA was first tested in Embase, and the search strategy subsequently adapted to the other databases. The bibliographies of included articles were evaluated for pertinent articles to ensure complete literature review.

### Selection criteria

Eligible study designs included randomized control trials, observational studies, and case series with at least 3 patients. We excluded case reports due to high risk of selection bias and lack of generalizability. We also excluded studies irrelevant to our topic, systematic reviews, biomechanical/cadaveric studies, and those published not in English where translation was unavailable.

The included study population was adult patients who developed ASF or scapular spine fracture post-rTSA. The included studies had to report on the type of management (operative or non-operative) used for post-rTSA ASF and qualitative and/or quantitative outcome measures. We did not limit studies based on setting, date of publication, indication for the primary rTSA, management protocol, or the length of follow-up for ASF post-rTSA.

### Screening

Two authors (A.A. and C.C.) independently performed title and abstract screening as well as full text review. Where consensus was not reached a third reviewer (D.N.) was invited to provide final decision. The search was carried out on December 26, 2025.

### Data extraction

The data extraction was performed by 2 reviewers (A.A. and C.C.). The data were extracted into an Excel spreadsheet. This included year of publication, study type, level of evidence, sample size, demographic characteristics, follow-up duration, location of fracture, imaging modality to diagnose fracture, management strategy, and clinic outcomes.

### Risk of bias

The methodological quality of the included studies was assessed using methodological quality of the included studies (Methodological Index for Non-Randomized Studies [MINORS]). The items are scored 0 (not reported), 1 (reported but inadequate), or 2 (reported and adequate). The maximum possible score is 16 points for non-comparative studies and 24 points for comparative studies.^25^ Quality of included studies was assessed independently (A.A. and C.C.). In cases where consensus was not reached, a third author (D.N.) was invited to reach final decision. The results of the quality assessment are summarized in Appendix 1.

### Analysis

Continuous variables were summarized using means and standard deviations (SDs) where reported, while categorical variables were expressed as absolute frequencies and percentages. Weighted mean and SDs, which takes consideration of the different sample sizes of studies, were calculated and presented when appropriate. Given the predominance of level III–IV evidence, variability in follow-up duration, and inconsistency in outcome definitions and reporting, formal meta-analysis and comparative effect size estimation were not undertaken. Accordingly, this study is a systematic review with descriptive quantitative synthesis rather than a meta-analysis. We pooled, where available, dichotomous outcomes across both treatment groups (union), between group proportions were compared using Chi-square test with a two-sided α of 0.05. Pooled patient-reported outcome measures were not compared statistically as the necessary patient level data and measures were inconsistently reported in the studies. Analyses were performed using SPSS (IBM SPSS Statistics; version 31.0.2.0; IBM, Armonk, NY, USA).

## Results

### Included studies

**Figure 1:**
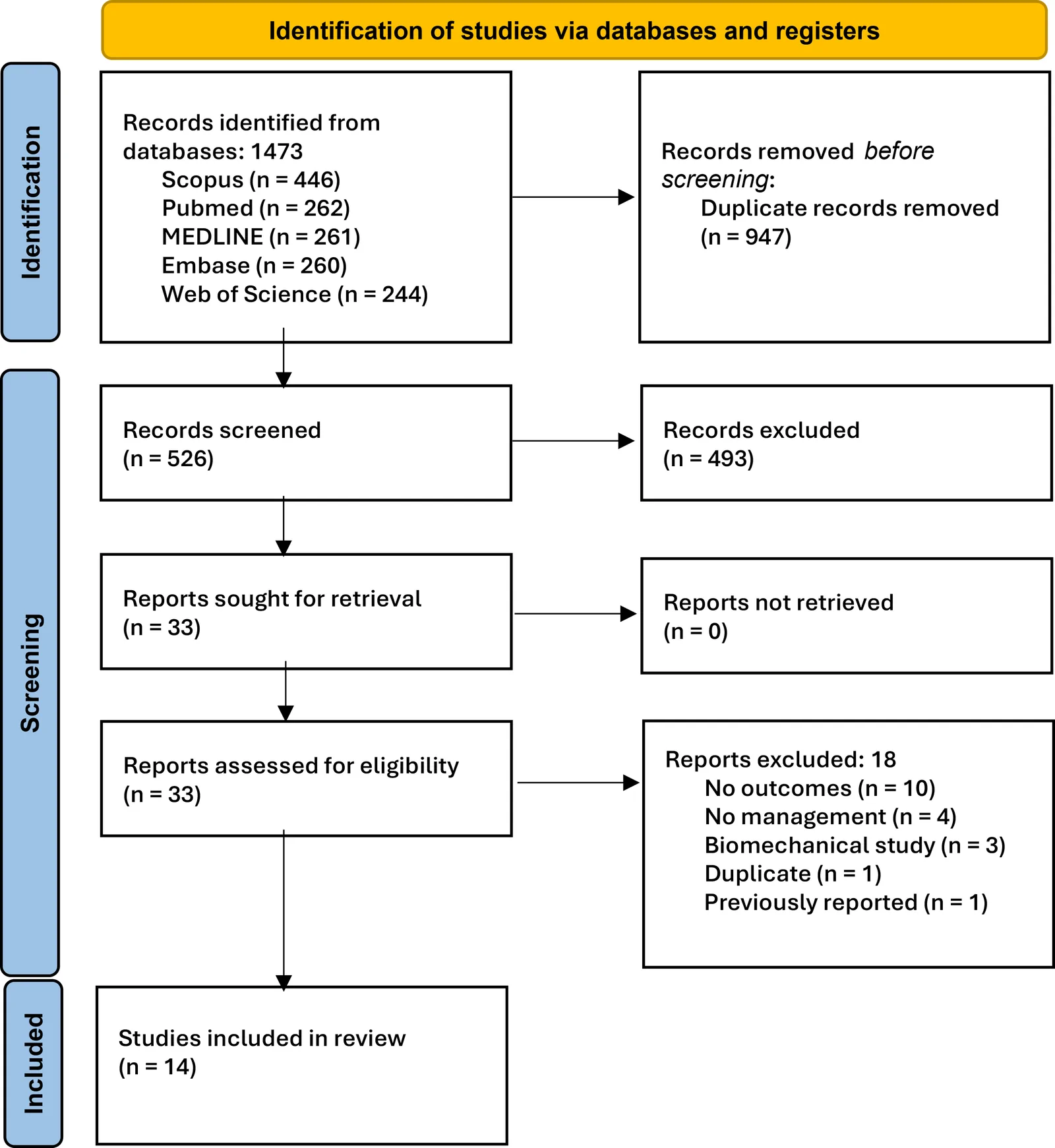
PRISMA flow diagram of the literature search. *PRISMA*, Preferred Reporting Items for Systematic Reviews and Meta-Analyses.

Fourteen studies were included (Fig. 1) and were published between 2010 and 2025.^2^^,^^8^^,^^9^^,^^11^^,^^15^^,^^21^, ^22^, ^23^, ^24^^,^^26^, ^27^, ^28^, ^29^^,^^31^ The risk of bias assessment of the included studies are presented in Appendix 1. The majority of the studies were level III-IV evidence (Table II). According to the MINORS criteria, the average score for the comparative studies (n = 7) is 16.4/24 and 8.9/16 for the non-comparative studies (n = 7).

**Table II:** Summary of the included studies.

Author	Year	Level of evidence	MINORS score	No. of patients	Age (mean)	Gender (M:F)	Incidence (%)	Prosthesis	Follow-up (mean/mo)
Bauer et al^2^	2025	III	16	74	75.3	14:60	NR	DJO Altivate (n = 7); n = 9 had Ascend Flex with perform reverse or Aqualis reverse II with BIO-rTSA (Stryker): DJO Altivate for non-op group.	43.9
Gutman et al^8^	2021	IV	15	27	71.8	7:20	4.5	NR	49.7
Hagelstein et al^9^	2025	IV	8	22	80	2:20	NR	Delta Xtend (Depuy) (n = 17). The others had revision elsewhere.	18
Hattrup^11^	2010	III	19	9	72.6	1:9	7.2	Delta III (Depuy) or Trabecular Metal Reverse Prostheses (Zimmer Biomet)	At least 12
Kriechling et al^14^	2022	III	17	44 (46 fractures)	74	10:34	5.4	Anatomical Shoulder Inverse/Reverse (Zimmer Biomet)	59
Moore et al^21^	2025	III	16	19	69.5	7:12	NR	Equinoxe (Exactech)	30
Neyton et al^22^	2019	IV	8	19	74	3:16	NR	Aequalis reversed (Tornier), Delta (DePuy); Aequalis Aequalis fracture long stem (Tornier)	97
Rhee et al^23^	2025	III	11	68	74	12:6	NR	NR	30
Schenk et al^24^	2022	III	15	30	72.3	8:22	NR	NR	33.8.
Teusink et al^26^	NR	III	17	25	72.2	4:21	3.1	Reverse Shoulder Prosthesis (DJO Surgical)	50
Toft et al^27^	2019	IV	10	5	76	5:0	NR	Universe Revers (Arthrex), PROMOS (Smith & Nephew), SMR Reverse Modular Shoulder System (Lima Corporate)	12
Tran et al.	2025	IV	7	11	70	4:7	NR	NR	13
Wahlquist et al^29^	2011	IV	8	5	74.2	1:5	NR	Delta (Depuy), Trabecular Metal Reverse Prostheses (Zimmer Biomet), Tornier	12-36
Yu et al^31^	2025	IV	10	16	66.1	10:5	NR	Zimmer Biomet; DePuy Synthes; Encore medical/DJO; Tornier/Viking; Arthrex	Minimum of 12

*MINORS*, Methodological Index for Non-Randomized Studies; *M*, male; *F*, female; *NR*, not reported; *BIO*-*rTSA*, Bony Increased Offset for Reversed Total Shoulder Arthroplasty.

### Demographic data

Three hundred seventy-six ASFs in 374 patients with a mean age of 72.9 years (6.8 SD) were studied, with 83 (22.2%) being male (Table II). The reported incidence of post-rTSA ASF was reported in 4 studies and ranged from 3.1 to 7.2%, this range should be interpreted with caution against the wider literature range of approximately 0.8% to 11%. Of the studies which reported the cause, 44 of 147 (29.9%) fractures were secondary to trauma, whereas the majority 70% (n = 103) occurred atraumatically. Across 10 studies including 259 patients, the mean time to diagnosis of ASF post-rTSA was 11.3 months (SD 15.5), using either radiograph or computed tomography scan (Table III). The mean follow-up duration after acromial fracture in 9 studies was 41.1 months (SD 20.8). Comorbidities were reported in 6 studies most common being diabetes (32.8%, n = 24), followed by osteoporosis (23.4%, n = 17), smoker (19.1%, n = 14), inflammatory arthropathy (5.5%, n = 4), cardiovascular condition (5.5%, n = 4), and asthma (2.7%, n = 2). Other comorbidities reported less commonly (each 1.4%, n = 1) include renal insufficiency, autoimmune condition, pulmonary condition, oral steroid use, adrenocortical insufficiency, anemia and Parkinson disease.

**Table III:** Characteristics of the included studies.

Author	Year	Fracture classification	Etiology (trauma:stress)	Time to fracture (mean/mo)	Time to surgery (mean/mo)	Diagnostic studies	Management	Outcomes reported
Bauer et al^2^	2025	Levy II (n = 39 non-op; n = 8 op). Levy III (n = 19 non-op; n = 8 op)	NR	NR	Within 1.4	Radiograph and CT scan	Operative (n = 16): 90-90 double plate (n = 11); Hook plating (n = 4); hybrid double construct (n = 1). Non-operative (n = 58): shoulder sling	Union, VAS pain, ASES, SSV, ROM
Gutman et al^8^	2021	Levy type I (n = 11); type 2 (n = 12); type 3 (n = 4)	NR	NR	NR	Radiograph ± CT scan	Non-operative: shoulder sling	Union, VAS, ASES, SANE, SST, ROM
Hagelstein et al^9^	2025	Seebauer classification mainly posterior medial fractures.	5:20	6	NR	Radiograph ± CT scan	Operative: double plating and autologous iliac crest bone graft	Union, ROM, complications
Hattrup^11^	2010	Acromion (n = 3) or scapular spine (n = 6)	5:4	10.9	NR	Radiograph	Non-operative: shoulder sling	VAS, ROM, ASES, SST, complications
Kriechling et al^14^	2022	Levy type 1 (n = 1), type 2 (n = 21), type 3 (n = 19)	9:37	16	3.5	Radiograph ± CT or MRI scan	Operative (n = 11): plate and/or tension band wiring. Non-operative (n = 35): shoulder sling or abduction brace	Union, CS, SSV, ROM, pain
Moore et al^21^	2025	Levy types I (n = 5 op; 1 non-op) type II (n = 2 op; n = 7 non-op); type 3 (n = 0 op vs. n = 4 non-op)	10:9	13	NR	Radiograph	Operative (n = 7): tension band constructs with plates/k-wires/cables. Non-operative (n = 12): shoulder sling	Union, VAS, ASES, SSV, ROM, complications
Neyton et al^22^	2019	Acromial base vs. scapular spine	3:16	5.34	NR	Radiograph ± CT scan	Non-operative: abduction brace	Union, ROM, CS
Rhee et al^23^	2025	Levy type I (n = 25), type II (n = 34), type III (n = 9)	NR	5.9		Radiograph	Non-operative: abduction brace	Union, VAS, ASES
Schenk et al^24^	2022	Crosby type 1 (n = 1 non-op; n = 3 op), type 2 (n = 15 non-op; n = 4 op), type 3 (n = 7 non-op)	NR	11.4	0.46	Radiograph ± CT scan	Operative (n = 7): tension banding, plate or both. Non-operative (n = 23): shoulder sling	Union, ROM, CS, SSV
Teusink et al^26^	NR	Acromion (n = 17), scapular spine fracture (n = 8).	NR	16	NR	Radiograph ± CT scan	Non-operative: shoulder sling	Union, ROM, ASES
Toft et al^27^	2019	Crosby type III (n = 5)	2:0	60	4.1	Radiograph and CT scan	Operative: double plating	VAS, CS, SPADI, SSV
Tran et al^28^	2025	Levy classification	4:7	NR	Unclear	NR	Operative: double or single plating, other constructs (screws, sutures, or wire) ± bone graft	Union, ROM
Wahlquist et al^29^	2011	Acromial base fractures (entire deltoid origin displaced)	NR	NR	NR	Radiograph ± CT scan	Operative (n = 3): single plate (n = 1), lag screw and tension band (n = 1), recon plate + bone grafting (n = 1). Non-operative (n = 2): Abduction brace	Union, pain, ROM, NFO, complications
Yu et al^31^	2025	Levy type 2 (n = 10), type 3 (n = 4)	6:10	10.6	11.4	Radiograph ± CT scan	Operative: double (n = 7) or single (n = 9) plating ± bone graft	Union, pain, ROM, SSV, ASES, PROMIS

*VAS*, visual analog scale; *ROM*, range of motion; *PROMIS*, Patient-Reported Outcomes Measurement Information System; *SANE*, Single Assessment Numeric Evaluation; *ASES*, American Shoulder and Elbow Surgeons; *SST*, Simple Shoulder Test; *SSV*, Subjective Shoulder Value; *NFO*, Neer functional outcomes; *SPADI*, Shoulder Pain and Disability Index; *CS*, Constant score; *CT*, computed tomography; *MRI*, magnetic resonance imaging.

Three classification systems were used in 10 studies namely Crosby, Levy, and Seebauer. Four studies (58 patients) described fracture location using descriptive terminology only (acromion or scapular spine). Levy was used more commonly (n = 7 studies), followed by Crosby (n = 2 studies) and Seebauer (n = 1). Among studies reporting Levy subtypes, type II was the most common (54.7%, n = 133), followed by type III (27.6%, n = 67) and type I (17.7%, n = 43). In studies employing the Crosby classification, type II fractures accounted for 54.3% (n = 19), followed by type III 34.3% (n = 12) and type I 11.4% (n = 25). In the single study utilizing, the Seebauer classification fractures were most commonly type III (63.6%, n = 14), followed by type II (27.2%, n = 6) and type III (9.1%, n = 2). The 4 studies that described fracture location without formal classification, acromial fractures were more common (60.3%, n = 35) than scapular spine fractures (39.7%, n = 23).

The most common indication reported across 10 studies for the primary rTSA was cuff tear arthropathy (47.9%; n = 140), followed by irreparable massive rotator cuff tear (27.0%; n = 79), glenohumerual osteoarthritis (10.3%, n = 30), revision from hemiarthroplasty or anatomic total shoulder arthroplasty (6.2%, n = 18), fracture (3.1%, n = 9), and previous failed cuff repair (2.4%, n = 7). Other less common indications include inflammatory arthropathy (1.03%, n = 3), instability (1.03%, n = 3), and AVN (1.03%, n = 3). A variety of reverse shoulder prosthesis was implanted prior to ASF. Nine included studies specified the rTSA prosthesis used (n = 217 patients). DJO/Enovis systems were reported for 90 patients (41.5%), followed by Zimmer/Zimmer Biomet platforms in 50 patients (23.0%) and DePuy Delta-lineage implants in 30 patients (13.8%). Stryker platforms including legacy Tornier/Wright Aequalis systems accounted for 23 patients (10.6%), while Exactech Equinoxe implants were used in 19 patients (8.8%). Few cases among the reported ASF featured Smith & Nephew PROMOS (3 patients, 1.4%), Arthrex Universe Reverse (1 patient, 0.5%), and LimaCorporate SMR (1 patient, 0.5%).

### Treatment

Ninety-eight patients (26.1%) underwent operative management, whereas 278 patients (73.9%) were treated non-operatively.

### Non-operative

Among the 10 studies that reported on non-operative management, treatment protocol typically involved shoulder immobilization using either sling (n = 6 studies) or an abduction brace (n = 4). The duration of immobilization ranged from 2 to 6 weeks, although 1 study reported use of abduction brace for 3 months. This was then followed by progressive physiotherapy.

### Operative: heterogeneity of fixation constructs and fracture patterns

A variety of surgical technique was reported for operative management of ASF post-rTSA. In the studies detailing fixation methods, double plating was most common (n = 52), followed by single plating (n = 15), hook plating (n = 4), and a hybrid double construct (n = 1). In 26 cases, breakdown of fixation method was not specified but included plate, tension band wiring, lag screw with tension band construct, or the use of k-wires and cables. Bone graft augmentation was used in select cases when deemed necessary. Only 2 operative series used a single uniform construct both double plating, every other series combined at least 2 construct types and did not report outcome union stratified by construct. Therefore, construct-specific union rate could not be reliably derived. Similarly, although operative cohort commonly featured Levy II and III fractures, union and functional outcomes were not commonly reported by fracture subtype, so outcomes could not be dissected by fracture pattern. These reporting gaps are why operative findings must be interpreted at the aggregate level and represent priority for standardized, construct, and subtype-specific reporting in future studies.

The interval from fracture diagnosis to surgical intervention varied considerably across the studies, which reported this parameter, ranging from <1.5 months to 11.4 months with reported ranges spanning from 0 to 48 months. Therefore, the surgical cohort was heterogenous not only in construct but also in fracture chronicity (acute, subacute to established non-unions). This could bias the operative group toward less favorable results.

### Outcomes reported

Outcomes for the included studies are based on those reported at the final follow-up after management of the ASF. More than half of the included studies did not report 1 or more of the patient-reported outcome measures, and the number of studies and/or patients contributing to each outcome is reported below.

### Union rates

The overall reported union rate following treatment of ASF post-rTSA was 68% (151/222) (Table IV). After conservative treatment, union rate was 59.8% (73/122). Conversely, the union rate after surgical fixation was 78% (78/100). The difference in pooled union rates between operative and non-operative treatment was statistically significant (Chi-square = 8.33, *P* = .004). This dichotomous outcome was only end point with sufficient data across both groups to permit statistical comparison, though should be interpreted cautiously due to it being based on pooled observation data with substantial heterogeneity.

**Table IV:** Outcomes of the included studies.

Author	Year	No. of patients	Intervention	Range of motion (°) Forward external elevation abduction rotation			Union (%)	VAS (mean)	ASES (mean)	Constant (mean)	SSV (mean)
Bauer et al^2^	2025	74	Operative (n = 16) Non-operative (n = 58)	Operative: 126 Non-operative: 96	Operative: NR Non-operative NR	Operative NR Non-operative: NR	Operative 94 Non-operative: 79	Operative 0 Non-operative: 2.5	Operative 82 Non-operative: 60	Operative NR Non-operative: NR	Operative 90 Non-operative: 67
Gutman et al^8^	2021	27	Non-operative	102	NR	25	53.6	NR	NR	NR	NR
Hagelstein et al^9^	2025	22	Operative	81.9	82.3	27.5	57	NR	NR	NR	NR
Hattrup^11^	2010	9	Non-operative	87.	79	36	11	4	24.4	NR	NR
Kriechling et al^14^	2022	44 patients (46 fractures)	Operative (n = 11) Non-operative (n = 35)	NR	NR	NR	Operative 82 Non-operative: 45	NR	NR	Overall 44	Overall 52
Moore et al^21^	2025	19	Operative (n = 7) Non-operative (n = 12)	Operative: 124 Non-operative: 134	Operative: NR Non-operative NR	Operative: 46 Non-operative: 46	Operative 71 Non-operative: 75	Operative 3.7 Non-operative: 1.8	Operative 62 Non-operative: 66	Operative NR Non-operative: NR	Operative NR Non-operative: NR
Neyton et al^22^	2019	19	Non-operative	109	NR	NR	42.1	NR	NR	47	NR
Rhee et al^23^	2025	68	Non-operative	NR	NR	NR	63	NR	NR	NR	NR
Schenk et al^24^	2022	30	Operative (n = 7) Non-operative (n = 23)	Operative: 75 Non-operative: 91.4	Operative: 67 Non-operative: 92.3	Operative: 13 Non-operative: 23.9	Operative 57 Non-operative: 30	Operative NR Non-operative: NR	Operative NR Non-operative: NR	Operative 33.2 Non-operative: 48.1	Operative 50 Non-operative: 58.3
Teusink et al^26^	NR	25	Non-operative	NR directly	NR directly	NR directly	55	NR	58	NR	NR
Toft et al^27^	2019	5	Operative	104	88	28	100	3	NR	44.4	60
Tran et al^28^	2025	11	Operative	106	NR	53	72.7	NR	NR	NR	NR
Wahlquist et al^29^	2011	5	Operative (n = 3) Non-operative (n = 2)	Operative: 55 Non-operative: 52.5	Operative: NR Non-operative: NR	Operative: NR Non-operative: NR	Overall: 100:	Operative NR Non-operative: NR	Operative NR Non-operative: NR	Operative NR Non-operative: NR	NR
Yu et al^31^	2025	16	Operative	91	NR	44	87.5	2	52	NR	44

VAS, visual analog scale; ROM, range of motion; ASES, American Shoulder and Elbow Surgeons; SSV, Subjective Shoulder Value; CS, Constant score; NR, not reported.

### Visual analog scale pain score

In the operative cohort, across 4 studies reporting visual analog scale pain score, the mean was 1.7 (SD 2.1) based on a total of 44 patients. In the non-operative group across the 2 studies, the mean was 2.4 (SD 3.5) based on a total of 70 patients.

### American Shoulder and Elbow Surgeons score

Among operative studies reporting ASES (n = 3 studies; 39 patients), the mean ASES score was 66.9 (SD 14.4). Whereas, in the non-operative group (n = 3 studies, 95 patients), the mean ASES score was 60.2 (SD 24.9).

### Subjective Shoulder Value score

Across the 4 operative studies that reported Subjective Shoulder Value, the mean was 63.5 (SD 17.9) based on a total of 44 patients. Whereas, in the non-operative cohort across 3 studies, the mean was 60.7 (SD 27.1) based on a total of 116 patients.

### Constant score

In the operative group, 2 studies that reported this Constant score had a mean of 37.9 (SD 13.0) based on a total of 12 patients. In the non-operative group across 3 studies, the mean was 46.0 (SD 20.8) based on a total of 77 patients.

### Range of motion

The mean external rotation in patients treated operatively across 6 studies (n = 68) was 36.0° (SD 27.4°). The mean shoulder external rotation achieved in the non-operatively treated patients (n = 62) was 28.7° (SD 16.0°), from the 3 studies that reported this outcome. The mean shoulder abduction in the operative group (n = 34) was 80.0° (SD 27.7°), whereas only 1 non-operative study (n = 23) reported this outcome with a mean of 92.3° (SD 39.5°). With regards to shoulder forward elevation, the operative group (n = 86), the mean shoulder forward elevation was 97.9° (SD 31.5°). In 6 non-operative studies (n = 141), the mean forward elevation was 100.8° (SD 42.5°).

### Complications

In the operatively managed ASFs post-rTSA, complications were reported in 28.2% of cases (22/78 patients), for whom this outcome was available. Reoperation was required in 91% of these cases (n = 20). The primary reasons for reoperation were hardware removal, most commonly due to symptomatic metalwork irritation in 60% (n = 12) and revision fixation, often related to hardware failure secondary to symptomatic non-union, in 30% (n = 6). Periprosthetic fractures occurring around the fixation construct accounted for the remaining 10% (n = 2). Additional complications that did not necessitate reoperation included screw displacement from the plate following fixation (n = 1) and iatrogenic pneumothorax resulting from drill or K-wire use (n = 1). Donor-site morbidity was also reported in selected studies utilizing autologous bone graft augmentation.

## Discussion

ASF post-rTSA represents a complex complication with multifactorial etiology and no universally accepted treatment algorithm. Although surgical fixation is associated with higher union rates, functional outcomes between non-operative and operative groups appear broadly comparable, with surgery carrying a notable complication profile. This review has confirmed consensus from existing literature, which demonstrates worse outcomes following ASFs post-rTSA irrespective of treatment modality.^5^^,^^16^ Several key themes emerge, which include low overall incidence of ASFs, 3.1 – 7.2%, among the 4 studies that reported it, similar to previous reports in the literature,^14^^,^^19^^,^^30^ but high functional impact of ASF, predominance of non-operative management strategies, the heterogeneity of surgical techniques, and persistent uncertainty with respect to optimal treatment pathways.

The observed demographic profile in this review is consistent with broader rTSA population with a mean age of 73 years and marked preponderance of female patients. This likely relates to both osteoporotic bone quality and epidemiology of cuff tear arthropathy, which may predispose to stress fractures. Most cases occurred without antecedent trauma suggesting fatigue failure due to biomechanical factors such as altered deltoid tensioning, stress concentration at scapular spine or acromion, and reduced bone mineral density playing a key role in ASF pathogenesis.^6^^,^^7^^,^^18^ The mean time to diagnosis approaching 1-year post-rTSA highlights the need for the ongoing clinical vigilance and lower threshold for advanced imaging in those presenting with worsening shoulder pain or functional decline after an initial satisfactory post-operative journey.

ASF classifications were variably applied, with the Levy system of which type II and III dominated. This distribution suggests that more medial fractures may be particularly susceptible following rTSA. It also reflects selection as Levy II and III fractures disrupt a greater proportion of deltoid origin they perhaps more often produce functional impartment and may preferentially be treated operatively, potentially introducing a confounding variable in operative and non-operative comparisons. However, heterogeneity in classification use and frequent reliance on descriptive terminology limited interstudy comparability. Therefore, future research would benefit from a uniform adoption of a validated system to enable more reliable stratification of outcomes by fracture subtype.

Non-operative management remains the most frequently employed strategy, utilized in almost 3 quarters of cases. This typically involved short-term shoulder immobilization followed by progressive physiotherapy. Although conservative management predominates, the mean union rate was about 60%, notably less than the 78% union rate achieved with surgical fixation (*P* = .004). This suggests while many patients may obtain acceptable symptom control without fixation, the risk of non-union remains substantial. Importantly, patient-reported outcome measures, including visual analog scale pain, ASES, and Subjective Shoulder Value scores, demonstrated only modest differences between treatment groups, although these differences were not formally tested owing to incomplete reporting, indicating radiographic union does not invariably translate into superior subjective outcomes. The discordance may perhaps, in part, reflect the relatively low functional demands of the older population studied or compensatory adaptation by surrounding musculature.

Operative management was associated with higher union rates but demonstrated a considerable complication burden. Wide variety of fixation methods were employed, most frequently double-plating constructs. The diversity in the surgical approach reflects absence of consensus regarding optimal fixation strategies to treat ASFs and the potential variability in surgeon-specific expertise related to scapular fixation techniques and a lack of optimally sized and angular stable plate constructs that are commercially available.^31^ Complication rates were approximately 30% with high proportion requiring reoperation most commonly for symptomatic hardware irritation or failure. These findings indicate while surgery may improve structural healing, it introduces additional morbidity, a factor that must be carefully weighed in the older or more medically frail patient population. A further source of heterogeneity is fracture chronicity. Operative cohorts pooled acute fractures, subacute, and established non-unions, while pragmatic non-unions and subacute fractures are biologically distinct from acute injuries and intrinsically less likely to heal. Hence, over-representation of theses may bias operative union and complication estimates unfavorably in the operative cohort. Our findings are broadly concordant with and extend 2 recent reviews in this area. Cassidy and colleagues^3^ focused specifically on fixation constructs and techniques likewise highlighting a preponderance of plate-based construct with an absence of comparative data. Isenberg et al,^12^ focused on operative management similarly reporting high union rates offset by substantial reoperation. By contrasting operative and non-operative management across union, patient-reported outcomes, and complications within a single dataset, the present review complements these fixation-focused analyses and reinforces the conclusion that current evidence base remains descriptive and hypothesis generating.

Range of motion reported outcomes were variable. Operative management showed a trend toward improved external rotation, whereas forward elevation was similar in both groups. The limited and inconsistent report of abduction and rotational shoulder parameters limited definitive conclusions. Furthermore, the small sample sizes and wide SD across the included studies indicates considerable interpatient variability.

This review deliberately compared operative with non-operative management of established ASF. It does not compare fractured with non-fractured rTSA patients, a distinct question addressed elsewhere and beyond our scope. Prior work reveals patients sustaining an ASF fare worse than those without, and the present data should be read as informing treatment selection once the fracture has occurred, not the overall prognostic impact of the fracture itself. More broadly the frequency and functional consequence of this complication argue for incorporating ASF risk into pre-operative counseling as well as ongoing scrutiny of rTSA indications, especially as indications continue to expand to younger or lower demand patients and to shoulders with intact or reparable cuffs, where alternative reconstructions may be considered.

Among the available literature, several limitations must be recognized. Firstly, this is a systematic review with descriptive synthesis, not a meta-analysis. Most of the included studies were evidence level III and IV with retrospective design and relatively small cohorts. More than half of the studies reported incomplete outcome data; due to this, pooled effect size estimation with formal statistical comparison was therefore possible only for union. Methodological assessment using the MINORS criteria was moderate at best with variable outcome reporting. An element of selection bias may be present as more severe or displaced fractures may have been treated surgically and a disproportionate number of non-unions and subacute injuries. Also, the absence of randomized or prospective comparative studies precludes firm causal inference regarding treatment superiority between the 2 groups. Furthermore, absence of randomized or prospective comparative studies precludes firm causal inferences regarding treatment superiority between the 2 groups and limits the strength of quantitative synthesis. Variability in follow-up duration, design of prosthesis, rehabilitation protocol, and outcome measures further limit strength of the pooled analyses.

While acknowledging above limitations, several practical implications can be drawn. Firstly, ASF post-rTSA is a clinically impactful complication that demands early recognition and careful longitudinal follow-up. Secondly, non-operative management remains appropriate for many patients, in particular those with minimally displaced fracture or limited functional demands, though clinicians should counsel patients appropriately regarding the higher non-union risk. Thirdly, surgical management may be considered for those displaced fractures or symptomatic non-unions with the understanding higher union rates must be balanced against increased complication and reoperation risks. There remains a clear requirement for prospective, multicenter studies using a standardized classification system with uniform outcome metrics to better define evidence-based management protocols.

## Conclusion

ASF post-rTSA constitutes a complex complication with multifactorial cause and no universally accepted treatment algorithm. While surgical fixation enhances union rates, functional outcomes between non-operative and operative groups remain broadly comparable with surgery being associated with notable complication profile. Individualized decision-making guided by fracture characteristics and patient-specific factors remains essential. The underlying evidence is Level IV and largely descriptive; these conclusions are hypothesis generating rather than definitive. Future prospective, comparative studies using standardized classifications, treatment methods, and outcome measures are essential to define clear indications for surgical intervention and to optimize clinical decision-making.

## Disclaimers:

Funding: No funding was disclosed by the authors.

Conflicts of interest: The author, their immediate family, and any research foundation with which they are affiliated have not received any financial payments or other benefits from any commercial entity related to the subject of this article.
