# Supplementary material for: Outcomes of operative and non-operative management of acromial and scapular spine fractures after reverse shoulder arthroplasty: a systematic review
Source: J Shoulder Elb Arthroplast. 2026 Aug 12;10(4):100081. doi: 10.1016/j.jsea.2026.100081 (PMC13594726; doi:10.1016/j.jsea.2026.100081)
Supplement: Supplementary file 1 [file mmc1.docx]

**Appendix 1: MINORS assessment of quality of included studies**

| Author | Clearly stated aim | Inclusion of consecutive patients | Prospective collection of data | Endpoints appropriate to aim | Unbiased assessment of endpoints | Follow-up appropriate to aim | Loss to follow up | Prospective calculation of study size | Comparative groups | Adequate control group | Contemporary groups | Baseline equivalence of groups | Adequate statistical analyses | MINORS score | Maximum possible score |
| --- | --- | --- | --- | --- | --- | --- | --- | --- | --- | --- | --- | --- | --- | --- | --- |
| Bauer et al.^2^ | 2 | 1 | 0 | 2 | 1 | 2 | 1 | 0 | Yes | 2 | 1 | 2 | 2 | 16 | 24 |
| Gutman et al.^8^ | 2 | 1 | 0 | 2 | 1 | 2 | 0 | 0 | Yes | 2 | 2 | 1 | 2 | 15 | 24 |
| Hagelstein et al.^9^ | 2 | 2 | 0 | 2 | 1 | 1 | 0 | 0 | No | - | - | - | - | 8 | 16 |
| Hattrup^11^ | 2 | 2 | 0 | 2 | 0 | 2 | 1 | 2 | Yes | 2 | 2 | 2 | 2 | 19 | 24 |
| Kriechling et al.^14^ | 2 | 2 | 0 | 2 | 1 | 2 | 0 | 0 | Yes | 2 | 2 | 2 | 2 | 17 | 24 |
| Moore et al.^21^ | 2 | 1 | 0 | 2 | 0 | 2 | 1 | 0 | Yes | 2 | 2 | 2 | 2 | 16 | 24 |
| Neyton et al.^22^ | 2 | 1 | 0 | 2 | 1 | 2 | 0 | 0 | No | - | - | - | - | 8 | 16 |
| Rhee et al.^23^ | 2 | 1 | 0 | 2 | 0 | 2 | 2 | 2 | No | - | - | - | - | 11 | 16 |
| Shenk et al.^24^ | 2 | 1 | 0 | 2 | 0 | 2 | 1 | 0 | Yes | 2 | 2 | 1 | 2 | 15 | 24 |
| Teusink et al.^26^ | 2 | 2 | 0 | 2 | 0 | 2 | 1 | 0 | Yes | 2 | 2 | 2 | 2 | 17 | 24 |
| Toft et al.^27^ | 2 | 2 | 0 | 2 | 0 | 2 | 2 | 0 | No | - | - | - | - | 10 | 16 |
| Tran et al.^27^ | 2 | 1 | 0 | 2 | 1 | 1 | 0 | 0 | No | - | - | - | - | 7 | 16 |
| Walquist et al.^29^ | 2 | 0 | 0 | 2 | 0 | 2 | 2 | 0 | No | - | - | - | - | 8 | 16 |
| Yu et al.^31^ | 2 | 2 | 0 | 2 | 1 | 2 | 1 | 0 | No | - | - | - | - | 10 | 16 |
